# Phase 1 pharmacokinetics and safety study of extended duration dapivirine vaginal rings in the United States

**DOI:** 10.1002/jia2.25747

**Published:** 2021-06-12

**Authors:** Albert Y Liu, Clara Dominguez Islas, Holly Gundacker, Blazej Neradilek, Craig Hoesley, Ariane van der Straten, Craig W Hendrix, May Beamer, Cindy E Jacobson, Tara McClure, Tanya Harrell, Katherine Bunge, Brid Devlin, Jeremy Nuttall, Patrick Spence, John Steytler, Jeanna M Piper, Mark A Marzinke, the MTN‐036/IPM 047 Protocol Team for the Microbicide Trials Network

**Affiliations:** ^1^ Bridge HIV San Francisco Department of Public Health San Francisco CA USA; ^2^ Department of Medicine University of California San Francisco CA USA; ^3^ Vaccine and Infectious Disease Division Fred Hutchinson Cancer Research Center Seattle WA USA; ^4^ Statistical Center for HIV/AIDS Research & Prevention Fred Hutchinson Cancer Research Center Seattle WA USA; ^5^ University of Alabama at Birmingham Birmingham AL USA; ^6^ Women’s Global Health Imperative (WGHI) RTI International Berkeley CA USA; ^7^ ASTRA Consulting Kensington CA USA; ^8^ Division of Clinical Pharmacology Department of Medicine Johns Hopkins University School of Medicine Baltimore MD USA; ^9^ Magee‐Womens Research Institute Pittsburgh PA USA; ^10^ FHI 360 Durham NC USA; ^11^ Department of Obstetrics, Gynecology, and Reproductive Sciences University of Pittsburgh Pittsburgh PA USA; ^12^ International Partnership for Microbicides Silver Spring MD USA; ^13^ Division of AIDS National Institutes of Health Bethesda MD USA

**Keywords:** dapivirine, vaginal ring, pharmacokinetics, safety, microbicide, pre‐exposure prophylaxis

## Abstract

**Introduction:**

Vaginal rings are a promising approach to provide a woman‐centred, long‐acting HIV prevention strategy. Prior trials of a 25 mg dapivirine (DPV) ring have shown a favourable safety profile and approximately 30% risk reduction of HIV‐1 infection. Extended duration rings replaced every three months may encourage user adherence, improve health service efficiency and reduce cost overall. We evaluated safety, pharmacokinetics, adherence and acceptability of two three‐month rings with different DPV dosages, compared with the monthly DPV ring.

**Methods:**

From December 2017 to October 2018, MTN‐036/IPM‐047 enrolled 49 HIV‐negative participant in Birmingham, Alabama and San Francisco, California into a phase 1, randomized trial comparing two extended duration (three‐month) rings (100 or 200 mg DPV) to a monthly 25 mg DPV ring, each used over 13 weeks, with follow‐up completed in January 2019. Safety was assessed by recording adverse events (AEs). DPV concentrations were quantified in plasma, cervicovaginal fluid (CVF) and cervical tissue, at nominal timepoints. Geometric mean ratios (GMRs) relative to the comparator ring were estimated from a regression model.

**Results:**

There were no differences in the proportion of participants with grade ≥2 genitourinary AEs or grade ≥3 AEs in the extended duration versus monthly ring arms (*p* = 1.0). Plasma and CVF DPV concentrations were higher in the extended duration rings compared to the monthly ring. Plasma GMRs were 1.31 to 1.85 and 1.41 to 1.86 and CVF GMRs were 1.45 to 2.87 and 1.74 to 2.60 for the 100 and 200 mg ring respectively. Cervical tissue concentrations were consistently higher in the 200 mg ring (GMRs 2.36 to 3.97). The majority of participants (82%) were fully adherent (ring inserted at all times, with no product discontinuations/outages) with no differences between the monthly versus three‐month rings. Most participants found the ring acceptable (median = 8 on 10‐point Likert scale), with a greater proportion of participants reporting high acceptability (9 or 10) in the 25 mg arm (73%) compared with the 100 mg (25%) and 200 mg (44%) arms (*p* = 0.01 and *p* = 0.15 respectively).

**Conclusions:**

The extended duration DPV rings were well‐tolerated and achieved higher DPV concentrations compared with the monthly DPV ring. These findings support further evaluation of three‐month DPV rings for HIV prevention.

## 1 Introduction

Over half of the 38 million people living with the human immunodeficiency virus (HIV) globally are women [1]. In sub‐Saharan Africa, women and girls account for 59% of new HIV infections, with young women being twice as likely to be living with HIV than men [2]. In the United States (US), nearly one‐fifth of new HIV diagnoses are among women, and Black women are particularly impacted [3].

While pre‐exposure prophylaxis (PrEP) is an effective approach to HIV prevention [4], adherence and persistence to daily oral PrEP and a daily or pericoital vaginal gel among women has been low across trials [5, 6, 7] and demonstration projects [8, 9]. Additionally, PrEP uptake has been slow among women, accounting for only 5% of US PrEP prescriptions [10]. These patterns illustrate significant barriers to the effectiveness of PrEP in women.

Antiretroviral‐based vaginal rings are a discreet, long‐acting HIV prevention approach that may provide an important alternative for women who are unable or choose not to use daily PrEP [11]. Dapivirine (DPV), a non‐nucleoside reverse transcriptase inhibitor with potent activity against HIV‐1, has been developed in a ring formulation [12]. Two phase 3 trials of a monthly 25 mg DPV ring among women in four African countries demonstrated a 27% to 35% overall reduction in risk of HIV infection [13, 14], and supportive data from two subsequent open‐label extension trials reported higher adherence and suggested higher effectiveness based on modelling [15, 16]. The European Medicines Agency (EMA) adopted a positive scientific opinion on the DPV ring under the Article 58 procedure (now EU‐Medicines4all) for use by adult cisgender women when oral PrEP is not/cannot be used or is unavailable, and the World Health Organization updated its clinical guidelines to include a recommendation for the monthly DPV ring as an additional choice for women as part of comprehensive prevention approaches. This is paving the road for its approval in countries where it is most urgently needed [17].

In a post hoc, non‐randomized analysis of the ASPIRE study, greater protection from viral acquisition was observed with more consistent ring use, as measured by residual DPV levels in used rings and plasma DPV concentrations [13, 18]. The development of a DPV ring with a higher loading dose is intended to extend the period of drug release, allowing for less frequent ring replacements, and may achieve higher local drug concentrations. Similar to contraceptive rings designed for use over multiple cycles [19, 20], extended duration rings for HIV prevention replaced quarterly may further reduce patient and provider burden and cost, increase accessibility, streamline follow‐up and improve adherence. The Microbicide Trials Network (MTN)‐036/International Partnership for Microbicides (IPM) 047 study evaluated the pharmacokinetics (PK), safety, adherence and acceptability of two extended duration rings loaded with 100 or 200 mg DPV for use over 13 weeks, as compared to the 25 mg monthly ring.

## 2 Methods

### 2.1 Study design

MTN‐036/IPM 047 was a phase 1 multi‐site, 3‐arm, randomized trial conducted at the University of Alabama at Birmingham (Birmingham, AL) and the San Francisco Department of Public Health (San Francisco, CA). Forty‐nine participants were enrolled in Birmingham (n = 25) and San Francisco (n = 24) between December 2017 and October 2018, with final study follow‐up completed in January 2019. Each site received local institutional review board approval.

The DPV rings are off‐white, flexible rings; all three rings had an outer diameter of 56 mm and a cross‐sectional diameter of 7.7 mm with DPV dispersed in a platinum‐cured silicone matrix and were visually identical. The comparator ring contained 25 mg DPV designed to provide sustained release over a minimum of one month, and the extended duration rings contained 100 or 200 mg DPV designed for sustained release over a minimum of three months.

The primary study objectives were to compare the safety and local and systemic PK of the extended duration rings to the comparator ring. Primary PK endpoints included DPV concentrations in plasma, cervicovaginal fluid (CVF) and cervical tissue. Safety was evaluated as the proportion of participants with grade ≥2 genitourinary adverse events (AEs) and grade ≥3 AEs, using the Division of AIDS Table for Grading the Severity of Adult and Pediatric Adverse Events and Female Genital Grading Table for Use in Microbicide Studies [21, 22]. Secondary objectives were to evaluate adherence and acceptability of the DPV rings. Adherence was assessed as the frequency and duration of self‐reported ring removal/expulsions captured by study staff on case report forms, and staff confirmed ring placement during study visits. Reasons for the ring outage were recorded. Acceptability was evaluated using a 10‐point Likert scale assessing the degree participants liked or disliked using the rings and the likelihood they would use the ring in the future if available. High acceptability was prespecified as having an acceptability score in the highest quintile (9 or 10). An exploratory objective included assessing residual DPV levels in returned rings.

Eligible participants were assigned female at birth, aged 18 to 45, HIV negative, using effective non‐ring‐based contraception, and generally healthy. Major exclusion criteria included: pregnant/breastfeeding; use of pre‐/post‐exposure HIV prophylaxis in the past three months; unresolved urinary or reproductive tract infection; sexually transmitted infection requiring treatment; chronic or recurrent candidiasis; significant hematologic or liver function test abnormalities; or clinically apparent grade ≥2 gynaecologic abnormalities.

After providing written consent and completing screening, eligible participants were randomized 1:1:1 to a 25 mg DPV ring (replaced every four weeks for eight weeks, then worn for five weeks), 100 mg DPV ring or 200 mg DPV ring (both used continuously for 13 weeks). The ring was inserted at enrolment, followed by a clinician‐performed exam to confirm placement. At each follow‐up visit, a clinician confirmed the correct placement of the ring by visualization with a speculum. Blood and CVF were collected one, two and four hours after ring insertion, and on day 91 immediately prior to ring removal, and one, two and four hours following ring removal. At all other study visits (days 1, 2, 3, 7, 14, 28, 56 and final contact on day 92 to 94), blood and CVF were obtained at a single timepoint for drug measurements. CVF was clinician‐collected via a vaginal swab within 30 minutes of blood collection, and the net weight of CVF was determined. Cervical tissue biopsies were collected at days 28 and 91 prior to ring removal; net biopsy weights were recorded, and biopsies were immediately flash‐frozen in a dry ice/ethanol bath. Used rings were collected for residual drug analysis at days 28 and 56 (monthly rings) and day 91 (all rings). Any severe or unexpected social harms were reported to the Protocol Safety Review Team.

### 2.2 Sample and randomization

The sample size of 48 participants was targeted, with 80% power to detect ~50% change in DPV concentrations (assuming coefficient of variation = 50%) [23]. The randomization scheme was generated and maintained by the MTN Statistical Data Management Center. Participants were randomized using permuted block randomization in a 1:1:1 ratio to the three study arms and were stratified by site to ensure balanced product assignment. A total of 49 participants were randomized.

### 2.3 Laboratory methods

DPV was quantified in plasma and cervical tissue using previously described, liquid chromatography‐tandem mass spectrometry (LC‐MS/MS) by the Clinical Pharmacology Analytical Laboratory at the Johns Hopkins University School of Medicine [24, 25]. A modified version of a previously published LC‐MS method, which employed CVF extraction from Dacron swabs via a 1:1 solution of methanol:water, was used for DPV quantitation in CVF [26]. All assays were validated in accordance with FDA bioanalytical guidelines. The lower limits of quantification (LLOQ) for DPV in plasma, CVF, and cervical tissue were 20 pg/mL, 0.250 ng/swab, and 0.05 ng/sample respectively. When normalized to fluid or biopsy weights, median LLOQs were 0.0036 ng/mg (interquartile range (IQR): 0.0028 to 0.0051 ng/mg) and 0.0027 ng/mg (IQR: 0.0019 to 0.0042 ng/mg) respectively. Residual DPV content in returned rings was determined by Pace Analytical Life Science (Oakdale, MN) using high‐performance liquid chromatography with photodiode array detection, as previously described [27]. Residual drug was evaluated separately for each monthly ring at day 28, 56 and 91 and for the three‐month rings at day 91.

### 2.4 Pharmacokinetic and statistical analysis

Participant characteristics were summarized using descriptive statistics. For the primary safety objective, which included all participants who inserted the ring, we compared the proportion of participants with AE endpoints in the extended duration ring arms relative to the monthly ring arm using Fisher’s exact test. Plasma, CVF and cervical tissue are reported for participants with >1 sample taken at or after day 28. The Area Under the Concentration–Time curves (AUC) in plasma and CVF were calculated for participants completing up to day 28 (AUC_0‐28d_) or day 91 (AUC_0‐91d_) visits, using the trapezoidal method. The peak concentration (C_max_) and time to peak concentration (T_max_) among participants completing up to the day 91 visit were also determined. To facilitate a fold increase comparison between arms, DPV concentration and exposure endpoints are summarized as geometric means (GMs) and geometric coefficients of variation (CV%), and geometric mean ratios (GMRs) were estimated from a fixed‐effects model on log‐transformed outcomes. To account for repeated measurements per participant, the model was fitted using Generalized Estimating Equations with an exchangeable correlation matrix. DPV concentrations reported as below the LLOQ were imputed with a value equivalent to half the LLOQ.

Participants were classified as fully adherent if they reported having kept the ring inserted at all times during the study, without any product discontinuation, hold or ring outage, except for ring changes in the monthly ring arm. Acceptability was assessed as the proportion of participants giving the ring a high score (9 or 10). Adherence and acceptability of the extended duration rings were compared with those of the monthly ring using Fisher’s exact test, and exact binomial 95% confidence intervals for the estimated proportions were calculated using Pearson–Clopper method. The mean and SD of the residual DPV levels in used rings were determined, along with the estimated total amount of DPV released over 91 days of study product use, calculated based on the manufacturer’s reported average load level in each ring batch. Additionally, unused rings (3 of each type) were analysed as a quality control check for the extraction process. All analyses were generated using SAS® and R software.

## 3 Results

**Figure 1:**
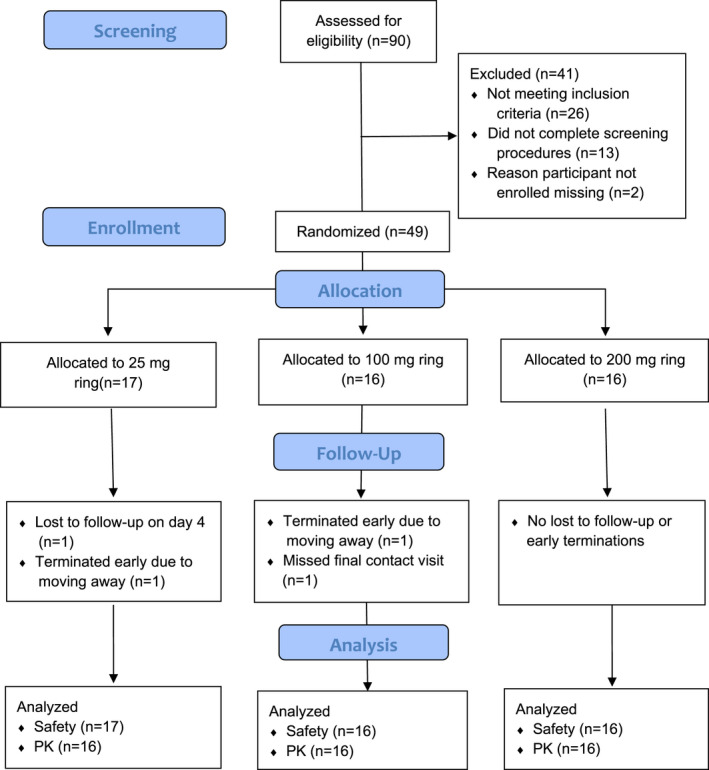
Flowchart of study participants.

Participant demographics and study flow are outlined in Table 1 and Figure 1 respectively. Four participants did not complete the study. In the 25 mg arm, one participant withdrew shortly after enrolment without a reason provided, and one participant relocated after day 56. In the 100 mg arm, one participant relocated after day 28, and one missed the final contact visit due to a family emergency. Two participants in the 200 mg arm refused cervical biopsy (one at both timepoints, one at day 91).

**Table 1:** Demographics and study‐related characteristics of participants in MTN‐036/ IPM 047 by study arm

	Comparator ring 25 mg DPV	Extended duration ring 100 mg DPV	Extended duration ring 200 mg DPV
N	17	16	16
Age, years			
Mean (SD)	30.5 (6.9)	30.6 (6.1)	29.0 (6.0)
Range	19, 44	19, 40	20, 40
Race			
Asian	2 (12%)	2 (13%)	0 (0%)
Black	8 (47%)	5 (31%)	7 (44%)
White	7 (41%)	5 (31%)	7 (44%)
Other	0 (0%)	4 (25%)	2 (13%)
Ethnicity			
Latina/Hispanic	0 (0%)	3 (19%)	2 (13%)
Gender identity			
Female	17 (100%)	15 (94%)	14 (88%)
Transgender	0 (0%)	0 (0%)	1 (6%)
Does not identify as male, female, or transgender	0 (0%)	1 (6%)	1 (6%)
Gender of sex partner(s)			
Male and female partners	2 (12%)	4 (25%)	2 (13%)
Exclusively female partners	2 (12%)	1 (6%)	3 (19%)
Exclusively male partners	11 (65%)	8 (50%)	9 (56%)
NA (no sex partners)	2 (12%)	3 (19%)	2 (13%)
Has ever used Vaginal Rings^a^			
Yes	6 (35%)	4 (25%)	1 (6%)
Number of participants with completed study visits, by visit			
Day 28 visit	16 (94%)	16 (100%)	16 (100%)
Day 56 visit	16 (94%)	15 (94%)	16 (100%)
Day 91 visit	15 (88%)	15 (94%)	16 (100%)
Final contact visit	15 (88%)	14 (88%)	16 (100%)

DPV, dapivirine; mg, milligram; N, Number; NA, Not applicable

^a^ Such as NuvaRing, Estring, Femring. As reported by participants at a baseline computer assisted self‐interview.

### 3.1 Safety Assessment

A total of 126 AEs were reported in 41/49 (84%) participants, including 40 AEs in 14/17 (82%) participants in the 25 mg arm, 47 AEs in 14/16 (88%) participants in the 100 mg arm and 39 AEs in 13/16 (81%) participants in the 200 mg arm. Overall, 30/126 (24%) of AEs were assessed as related to study product. The most commonly reported related AEs included vaginal discharge, uterine spasm, metrorrhagia and vaginal odour. Most AEs were grade 1 (99/126 [79%]) or grade 2 (26/126 [21%]), with one grade 3 AE reported in the 25 mg arm (intussusception, unrelated to study product). While all grade ≥2 genitourinary AEs were assessed as related, we found no statistically significant difference in the proportion of participants with grade ≥2 genitourinary AEs in the 100 mg (1/16 [6%]) or 200 mg (1/16 [6%]) arms compared with the 25 mg arm (2/17 [12%]), *p* = 1.00 for both comparisons. We also found no statistically significant difference in the proportion of participants with grade ≥3 AEs in the 100 mg (0/16 [0%]) or 200 mg (0/16 [0%]) arms compared with the 25 mg (1/17 [6%]) arm, *p* = 1.00. No severe or unexpected social harms were noted in the study.

### 3.2 Pharmacokinetic analysis

GM DPV concentrations in plasma, CVF, and cervical tissue are summarized in Table 2. Across different timepoints, the GMs of plasma DPV concentrations were 1.3 to 1.8 times higher in the 100 and 200 mg arms compared with the 25 mg arm. Additionally, DPV plasma C_max_ and AUC_(0‐28d)_ were nearly two‐fold higher for the extended duration ring. GMs of CVF DPV concentrations were 1.5 to 2.9 times higher in the extended duration ring arms, and C_max_ and AUC(_0‐28d_) were approximately 1.5 and 2‐fold higher, respectively, for the extended duration versus monthly ring. Compared with the 25 mg ring, GMs for cervical tissue DPV concentrations were higher in the 200 mg arm at day 28 and higher in both extended duration ring arms at day 91.

**Table 2:** Dapivirine concentrations in plasma (pg/mL), cervicovaginal fluid (ng/mg) and cervical tissue (ng/mg)

	Plasma			CVF			Cervical tissue		
	25 mg ring (n = 16)	100 mg ring (n = 16)	200 mg ring (n = 16)	25 mg ring (n = 16)	100 mg ring (n = 16)	200 mg ring (n = 16)	25 mg ring (n = 16)	100 mg ring (n = 16)	200 mg ring (n = 16)^a^
Day 28 visit									
Geometric mean, CV%	221, 30%	409, 25%	411, 38%	13, 88%	24, 92%	23, 129%	1.59, 143%	1.57, 1639%	3.76, 153%
GMR (95% CI)^c^	–	1.85 (1.54 to 2.22)	1.86 (1.48 to 2.32)	–	1.90 (1.13 to 3.18)	1.83 (1.01 to 3.29)	–	0.99 (0.29 to 3.37)	2.36 (1.13 to 4.91)
Day 56 visit									
Geometric mean, CV%	233, 35%	339, 31%	324, 127%^b^	8, 377%	24, 72%	21, 132%			
GMR (95% CI)^c^	–	1.45 (1.16 to 1.81)	1.41 (0.86 to 2.31)^b^	–	2.87 (1.21 to 6.85)	2.60 (1.01 to 6.67)			
Day 91 visit									
Geometric mean, CV%	220, 35%	276, 26%	315, 40%	11, 93%	14, 110%	17, 141%	0.70, 1065%	2.40, 336%	2.97, 176%
GMR (95% CI)^c^	–	1.31 (1.05 to 1.64)	1.45 (1.14 to 1.86)	–	1.45 (0.75 to 2.79)	1.74 (0.90 to 3.36)	–	3.04 (0.80 to 11.54)	3.97 (1.15 to 13.76)
Day 91 visit, 4 hours after ring removal									
Geometric mean, CV%	217, 36%	290, 37%	308, 43%	6.5, 110%	7.9, 259%	9.3, 123%			
Geometric Mean, CV% of fold change (relative to before ring removal)	0.98, 9%	0.97, 14%	0.98, 9%	0.56, 54%	0.54, 94%	0.54, 53%			
C_max_									
Geometric mean, CV%	280, 32%	487, 25%	505, 30%	33, 76%	48, 70%	51, 80%			
GMR (95% CI)^d^	–	1.74 (1.41 to 2.15)	1.81 (1.47 to 2.22)	–	1.48 (0.90 to 2.42)	1.56 (0.96 to 2.53)			
T_max_, days									
Geometric Mean, CV%	25, 128%	19, 77%	16, 57%	1.0, 729%	6.7, 563%	5.2, 619%			
GMR (95% CI)^d^	–	0.76 (0.44 to 1.33)	0.65 (0.38 to 1.12)	–	6.46 (1.56 to 26.73)	5.00 (1.24 to 20.23)			
AUC (0 to 28), pg/mL × days for plasma, ng/mg × days for CVF									
Geometric mean, CV%	6222, 33%	11143, 29%	11567, 28%	411, 80%	786, 114%	806, 114%			
GMR (95% CI)^e^	–	1.79 (1.45 to 2.21)	1.86 (1.50 to 2.30)	–	1.91 (1.04 to 3.50)	1.96 (1.07 to 3.59)			
AUC (0 to 91), pg/mL × days for plasma, ng/mg × days for CVF									
Geometric Mean, CV%	–	33249, 24%	34780, 39%	–	2154, 77%	2268, 109%			
GMR (95% CI)^f^	–	5.34 (4.24 to 6.73)	5.59 (4.45 to 7.02)	–	5.23 (2.97 to 9.21)	5.51 (3.19 to 9.52)			

CVF, cervicovaginal fluid; mg, milligram; CV, coefficient of variation; GMR, geometric mean ratio; CI, confidence interval; C_max_, peak concentration; T_max_, time to peak concentration; AUC, area under the concentration–time curve from 0 to 28 or 0 to 91 days; pg/mL, picograms per millilitre

^a^ Of 16 participants, one did not provide cervical tissue biopsy at Day 28 visit and two did not provide biopsies at Day 91 visit

^b^ The geometric mean, CV% and geometric mean ratio at this timepoint are highly influenced by one observation with DPV concentration reported as BLQ. No ring outages were reported by this participant. A sensitivity analysis excluding this observation yielded a geometric mean concentration of 409 pg/mL (CV: 34%) for participants in the 200 mg DPV arm, and a geometric mean ratio of 1.69 (95% CI: 1.32 to 2.16) relative to the comparator ring

^c^ All GMRs reflect comparisons with the 25 mg ring

^d^ Excludes two participants, in the 25 mg and the 100 mg ring arms, who discontinued the study before the last product use visit at Day 91

^e^ GMRs reflect comparison to the AUC (0 to 28) in the comparator ring group (excludes one participant in the 100 mg ring arm who discontinued the study before the last product use visit at Day 91).

**Figure 2:**
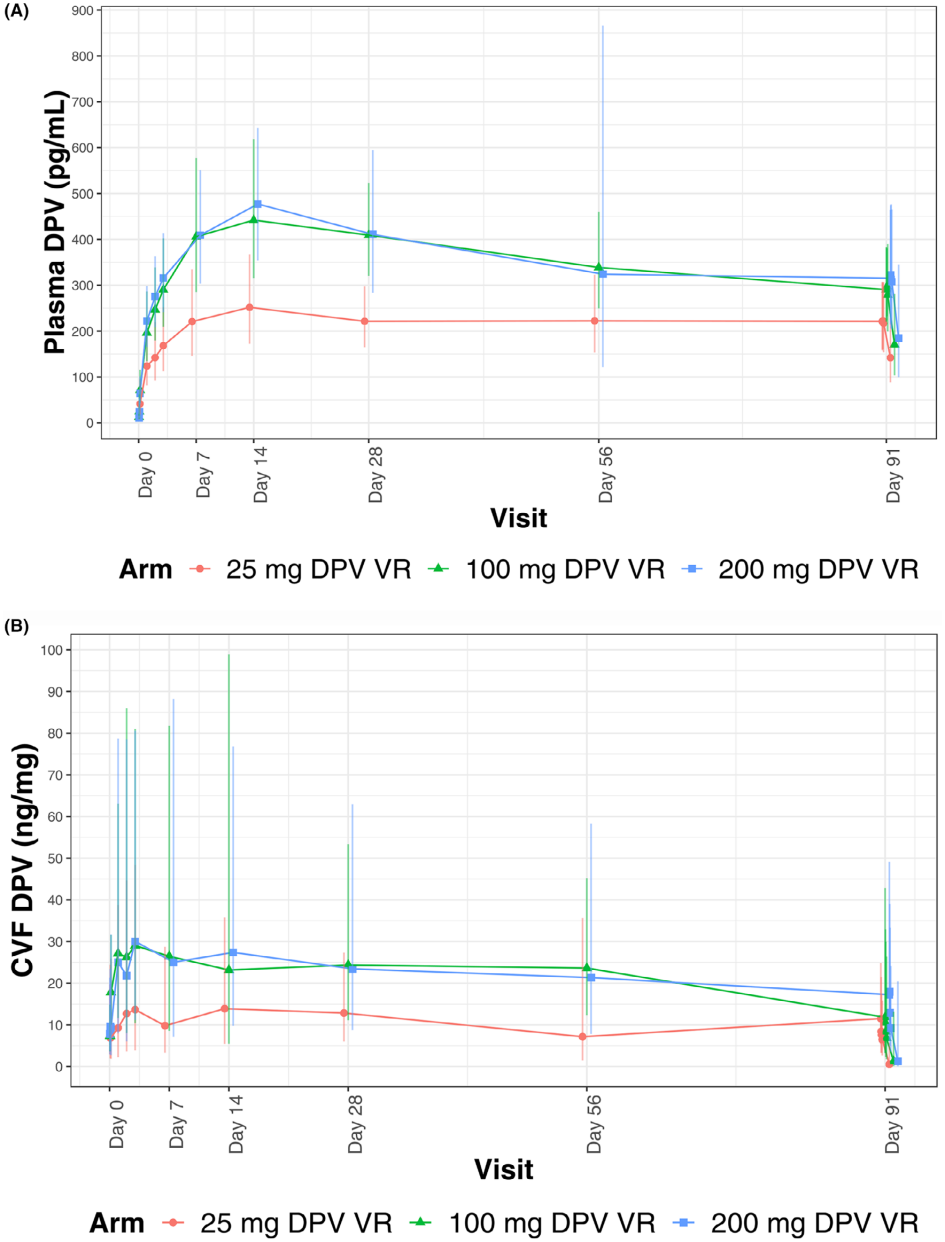
Geometric means of DPV concentration in **(A)** blood plasma (pg/mL) and **(B)** CVF (ng/mg). Vertical bars indicate the back transformation of [Mean ± 1 SD] intervals of log‐transformed concentrations.

Concentration–time curves for DPV in plasma and CVF are shown in Figure 2. The GM T_max_ ranged from sixteen to twenty‐five days in plasma and one to seven days in CVF, with a longer T_max_ for the extended duration rings observed in CVF but not plasma. GM DPV concentrations remained similar four hours after ring removal in plasma, but dropped by about half in CVF, when compared with DPV concentrations prior to ring removal.

### 3.3 Adherence, Residual DPV Ring Levels and Acceptability

Most participants (40/49, 82%) reported being fully adherent during the study: 76% (95% CI 50% to 93%) in the 25 mg arm, 81% (95% CI 54% to 96%) in the 100 mg arm and 88% (95% CI 62% to 98%) in the 200 mg arm, with no statistically significant differences between groups. Among nine participants who were not fully adherent, three terminated early; three reported a single outage, two reported two outages and two reported three outages. Three participants reported ring outages >12 hours continuously. Reported reasons for ring removal included discomfort or other symptoms, menses/bleeding, to clean the ring, not wanting the partner to know about ring, partner disliking the ring and/or wanting it removed and removal for sex or pelvic exam. Two ring expulsions were reported, once from tampon removal and once related to sex.

Mean residual DPV levels in used rings were 20.9, 21.1 and 20.5 mg at days 28, 56 and 91 in the 25 mg arm, and 86.7 and 184.3 mg in the 100 and 200 mg arms, respectively, at day 91. Based on manufacturer‐reported averaged DPV loads (24.5, 100.5 and 207.1 mg/ring for the 25, 100 and 200 mg rings respectively), the mean total DPV released over 13 weeks was estimated to be 11.2 mg (range 7.4 to 16.1) for the 25 mg ring, 14.2 mg (10.2 to 20.4) for the 100 mg ring and 22.8 mg (12.8 to 28.5) for the 200 mg ring. The mean DPV concentrations in unused control rings (N = 3) were lower than the batch concentrations reported by the manufacturer (N = 10), particularly for the 200 mg ring (201.0 vs. 207.1 mg), suggesting some minor variability in the assay and/or ring loading.

**Figure 3:**
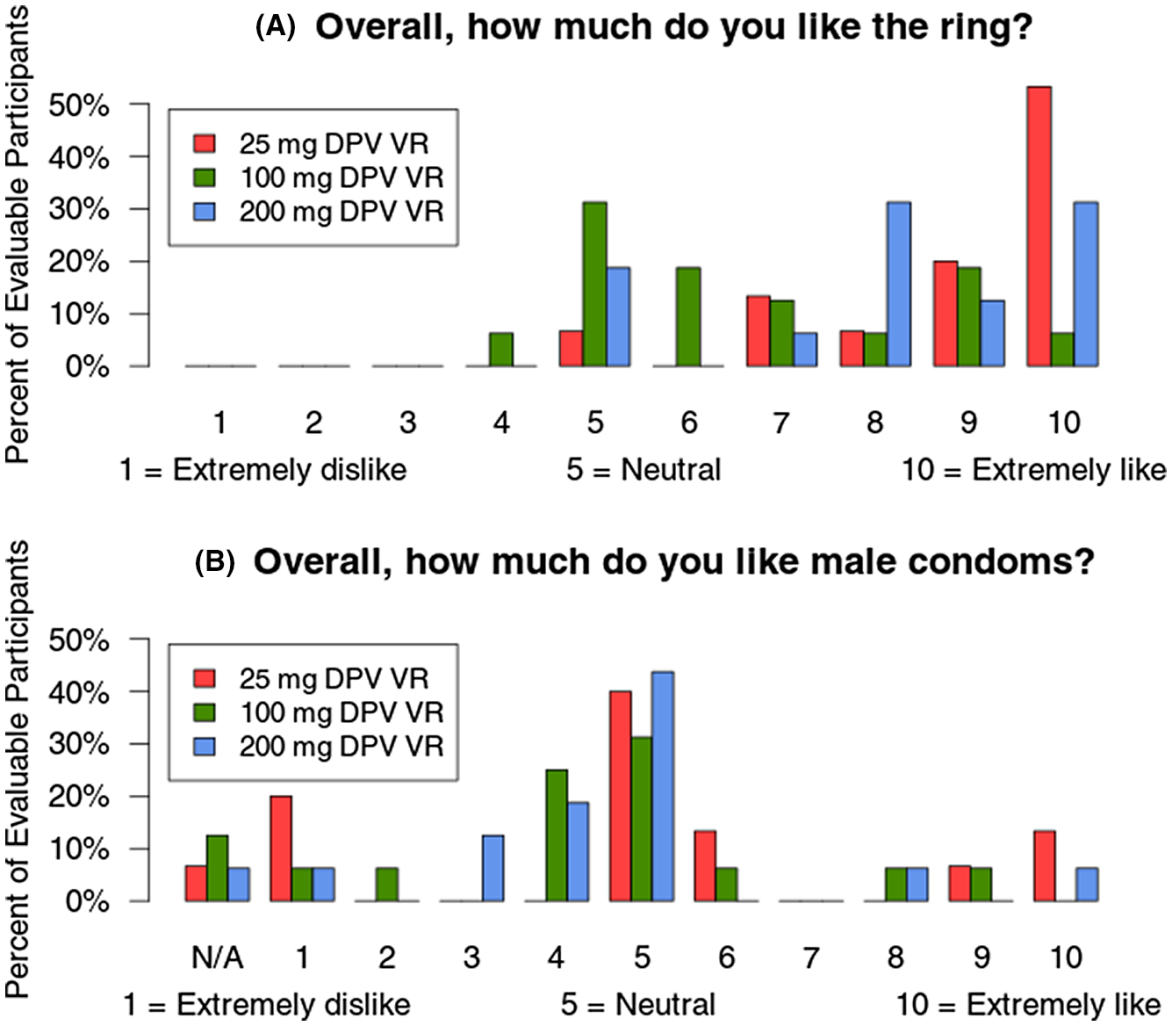
Acceptability of comparator 25 mg and extended duration 100 and 200 mg rings **(A)** in relation to male condoms **(B)**. The choice “Never used (N/A)” was allowed for male condoms but not for the ring.

Acceptability of the rings at the study exit was high. When using the pre‐specified cut‐point, acceptability was higher in the 25 mg ring, with 73% of participants giving a score of 9 or above, compared with 25% in the 100 mg arm (*p* = 0.01) and 44% in the 200 mg arm (*p* = 0.15) (Figure 3A). However, only one participant gave a score below 5 = neutral (100 mg DPV arm, Figure 3A), and acceptability scores for the rings (median: 8 (IQR 6 to 10)) compared favourably to condoms (median: 5 (IQR 4 to 5)) (Figure 3B). Scores were similar among those who had previously used rings (median 8 (IQR 5 to 10)) and those who did not (median 8 (IQR 6.5 to 10)). When asked how likely participants would be to use the ring if effective, most gave scores above 5, indicating likelihood of future use (median: 9 (IQR 7 to 10)).

## 4 Discussion

In this study evaluating two three‐month DPV rings, the 100 and 200 mg DPV rings were found to be well‐tolerated when compared with the monthly 25 mg ring, with no safety concerns identified. The safety profile of the extended duration rings was similar to the 25 mg ring, which has been demonstrated to be well‐tolerated in prior trials [23, 25, 28, 29, 30, 31].

We found that, on average, plasma and CVF concentrations, as well as AUC(_0‐28d_), were higher in the 100 and 200 mg ring arms compared with the 25 mg ring arm. Concentration–time curves showed a rapid rise in DPV concentrations, which were similar across arms the first day after insertion, but the extended duration rings subsequently achieved higher C_max_, on average. The T_max_ in plasma tended to be shorter for the extended duration rings, while the T_max_ in CVF was longer, relative to the comparator ring. As GM concentrations of DPV in vaginal fluid declined rapidly after ring removal in all three rings, it is recommended that the rings be kept in place continuously, including during menses. Both extended duration rings had higher cervical tissue concentrations at day 91 (only 200 mg ring had higher tissue concentrations at day 28). Even if the rate of DPV concentration decline in tissue is less rapid than in CVF, temporary removal of the three‐month rings likely results in concentrations for many participants falling below typical concentrations seen for the inserted 25 mg ring – our benchmark for comparative efficacy. While most PK endpoints tended to be higher in the 200 versus 100 mg arm, there were no statistically significant differences observed. The highest GM concentration in plasma associated with any of the rings was 505 pg/mL (C_max_ for the 200 mg ring) which was more than 4500‐fold lower than the mean maximum concentration (2286 ng/mL) observed at the maximum tolerated oral dose of DPV [23, 30]. Of note, oral DPV is not a formulation being pursued for HIV prevention. While DPV concentrations in fluids and tissue were not dose proportional, there was a 27% to 103% greater DPV release from the extended duration versus comparator rings, resulting in approximately 1.5 to three‐fold increase in plasma, CVF, and cervical tissue concentrations. These findings support the use of these extended duration DPV rings for up to 13 weeks. Although the PK threshold for efficacy for dapivirine remains unknown, it is expected that equal or higher DPV concentrations achieved across compartments at all time points over the course of the use cycle with the extended duration rings should translate into equal or higher efficacy as compared to the monthly ring. As plasma and vaginal fluid concentrations at day 91 for the three‐month rings were approaching levels seen on day 91 with the third 25 mg ring, the use period for these extended duration formulations will likely be limited to 90 days. An additional bioavailability study is being planned to further characterize the PK of the three‐month rings relative to the 25 mg ring to inform future development.

Adherence was high for all three rings, with no differences across arms observed. The mean residual DPV in rings for the 25 mg arm (20.5 to 21.1 mg) suggested adherence to ring use, based on prior studies and benchmarks used to assess adherence (residual DPV ≤23.5 mg) [13, 29]. While ring removals were infrequent, several participants reported ring outages due to partner‐related concerns. Prior studies have highlighted the important role of male partners on ring use and acceptability [32, 33, 34]. A few participants in each arm reported removing the ring during menses. These concerns have been raised in previous studies in which women reported removing the ring during menses for cleaning, concerns the ring would block menstrual blood flow and menstrual pain attributed to the ring [35, 36, 37]. Education and support to address partner‐related concerns and ring use during menses will be important for these rings.

Ring acceptability was high across arms, with somewhat higher acceptability ratings for the monthly versus extended duration rings. This finding may reflect greater initial familiarity with the monthly ring, as evidenced by higher baseline acceptability ratings with the monthly versus extended duration rings [38]. Despite higher acceptability ratings with the monthly ring, most participants in this study reported preference for the three‐month rings at study exit due to increased convenience, although preferences varied by site, education, race/ethnicity and prior ring use experiences [38]. Importantly, acceptability to novel prevention technologies tends to increase over time with greater use [39, 40]. Most participants were interested in using the ring in the future.

This study has several limitations. First, measured variability of dapivirine in post‐use rings is high, likely due to unknown, patient‐specific factors (actual use time, vaginal environment, biological fluids, etc.). Secondary factors which could also influence the measured results are analytical method variability (pre‐ and post‐use), and to a smaller extent, manufacturing variability. Second, as DPV concentrations were measured monthly after day 28, AUC (_0‐91d_) could only be calculated for the extended duration rings. Additionally, social desirability may have impacted assessments for self‐reported adherence and acceptability measures. This study also had a number of strengths, including enrolling a diverse cohort across two geographically distinct sites, the comparison of two extended duration rings to the monthly ring, and extended follow‐up for 13 weeks.

## 5 Conclusions

In summary, in this Phase I study, the extended duration DPV rings were found to be well‐tolerated, with comparable safety findings between groups and high rates of adherence as well as good acceptability. PK findings demonstrate higher DPV concentrations achieved in plasma, CVF and cervical tissue with the extended duration rings and provide robust support for continued development of three‐month rings. If approved, these extended use rings could provide women with additional long‐acting options and further increase access and equity to HIV prevention in women globally.

## Competing interests

AL has received funding for investigator‐sponsored research grants from Gilead Sciences and Viiv Healthcare, and has led studies in which Gilead Sciences has donated study drugs. AVS has led preclinical studies in which Gilead Sciences has donated study drug. CWH has received funding for investigator‐sponsored research from and served on ad hoc scientific advisory boards for Gilead Sciences, ViiV/GSK, and Merck. CDI, HG, BN, CH, MB, CEJ, TM, TH, KB, BD, JN, PS, JS, JP and MM declare that they have no competing interests.

## AUTHOR’S CONTRIBUTIONS

AL, CDI, HG, CH, AVS, CWH, MB, CEJ, TM, TH, BD, JN, PS, JS, JP and MM assisted with protocol development and design of the analysis. AL and CH collected the data. CDI, HG, BN, MB, TH and MM conducted the analyses. AL and CDI wrote the paper. PS contributed to the residual DPV work and discussion. All authors reviewed, edited and approved the final manuscript.

## References

[1] UNAIDS . Women and HIV. A spotlight on adolescent girls and young women [cited 2020 Jul 29]. Available from: https://www.unaids.org/sites/default/files/media_asset/2019_women‐and‐hiv_en.pdf

[2] UNAIDS . Global HIV & AIDS statistics – 2020 fact sheet [cited 2020 Jul 29] https://www.unaids.org/en/resources/fact‐sheet

[3] Centers for Disease Control and Prevention . HIV and women [cited 2020 Jul 29]. Available from: https://www.cdc.gov/hiv/group/gender/women/index.html

[4] Hodges‐Mameletzis I , Fonner VA , Dalal S , Mugo N , Msimanga‐Radebe B , Baggaley R . Pre‐exposure prophylaxis for HIV prevention in women: current status and future directions. Drugs. 2019;79(12):1263–76. 31309457 10.1007/s40265-019-01143-8

[5] Marrazzo JM , Ramjee G , Richardson BA , Gomez K , Mgodi N , Nair G , et al. Tenofovir‐based preexposure prophylaxis for HIV infection among African women. N Engl J Med. 2015;372(6):509–18. 25651245 10.1056/NEJMoa1402269 PMC4341965

[6] Van Damme L , Corneli A , Ahmed K , Agot K , Lombaard J , Kapiga S , et al. Preexposure prophylaxis for HIV infection among African women. N Engl J Med. 2012;367(5):411–22. 22784040 10.1056/NEJMoa1202614 PMC3687217

[7] Delany‐Moretlwe S , Lombard C , Baron D , Bekker L‐G , Nkala B , Ahmed K , et al. Tenofovir 1% vaginal gel for prevention of HIV‐1 infection in women in South Africa (FACTS‐001): a phase 3, randomised, double‐blind, placebo‐controlled trial. Lancet Infect Dis. 2018;18(11):1241–50. 30507409 10.1016/S1473-3099(18)30428-6

[8] Celum C , Mgodi N , Bekker L‐G , Hosek S , Donnell D , Anderson PL , et al. PrEP use in young African women in HPTN 082: Effect of drug level feedback. Paper presented at: 10th IAS Conference on HIV Science; 2019 Jul 21‐24; Mexico City, Mexico. Abstract TUAC0301.

[9] Blumenthal J , Jain S , He F , Rivet Amico K , Kofron R , Ellorin E , et al. Results from a PrEP demonstration project for at‐risk cisgender women in the US. Paper presented at: Conference on Retroviruses and Opportunistic Infections; 2020 Mar 8‐11; Boston. Abstract 1036.

[10] Huang YA , Zhu W , Smith DK , Harris N , Hoover KW . HIV preexposure prophylaxis, by race and ethnicity ‐ United States, 2014–2016. MMWR Morb Mortal Wkly Rep. 2018;67(41):1147–50. 30335734 10.15585/mmwr.mm6741a3 PMC6193685

[11] Spence P , Bhatia Garg A , Woodsong C , Devin B , Rosenberg Z . Recent work on vaginal rings containing antiviral agents for HIV prevention. Curr Opin HIV AIDS. 2015;10(4):264–70. 26049952 10.1097/COH.0000000000000157

[12] Devlin B , Nuttall J , Wilder S , Woodsong C , Rosenberg Z . Development of dapivirine vaginal ring for HIV prevention. Antiviral Res. 2013;100 Suppl:S3–8. 24188702 10.1016/j.antiviral.2013.09.025

[13] Baeten JM , Palanee‐Phillips T , Brown ER , Schwartz K , Soto‐Torres LE , Govender V , et al. Use of a vaginal ring containing dapivirine for HIV‐1 prevention in women. N Engl J Med. 2016;375(22):2121–32. 26900902 10.1056/NEJMoa1506110 PMC4993693

[14] European Medicines Agency . Dapivirine Vaginal Ring 25 mg: Medicine overview. 2020. https://www.ema.europa.eu/en/dapivirine‐vaginal‐ring‐25‐mg‐h‐w‐2168

[15] Baeten J , Palanee‐Phillips T , Mogodi N , Ramjee G , Gati B , Mhlanga F , et al. High adherence and sustained impact on HIV‐1 incidence: Final results of an open‐label extension trial of the dapivirine vaginal ring. Paper presented at: 10th IAS Conference on HIV Science; 2019 Jul 21‐24; Mexico City, Mexico. Abstract TUAC0203.

[16] Nel A , Niekerk NV , Baelen BV , Rosenberg Z . Safety, Adherence and HIV‐1 Seroconversion In DREAM – An Open‐Label Dapivirine Vaginal Ring Trial. Paper presented at: 9th South African AIDS Conference; 2019 Jun 11‐14; Durban, South Africa.

[17] National Institute of Allergy and Infectious Diseases . Vaginal Ring for HIV Prevention Receives Positive Opinion from European Regulator: NIAID Celebrates Pivotal Step Toward Expanding HIV Prevention Choices for Women. 2020 [cited 2020 Aug 6]. https://www.niaid.nih.gov/news‐events/vaginal‐ring‐hiv‐prevention‐receives‐positive‐opinion‐european‐regulator#qa‐section

[18] Brown E , Palanee‐Philips T , Marzinke M , Hendrix C , Dezutti C , Soto‐Torres L , et al. Residual dapivirine ring levels indicate higher adherence to vaginal ring is associated with HIV‐1 protection. Paper presented at: AIDS 2016; 2016 Jul 18‐22; Durban, South Africa. Abstract TUAC0105LB.

[19] Archer DF , Merkatz RB , Bahamondes L , Westhoff CL , Darney P , Apter D , et al. Efficacy of the 1‐year (13‐cycle) segesterone acetate and ethinylestradiol contraceptive vaginal system: results of two multicentre, open‐label, single‐arm, phase 3 trials. Lancet Global health. 2019;7(8):e1054–64. 31231065 10.1016/S2214-109X(19)30265-7 PMC6624423

[20] Temmerman M . A new woman‐controlled contraceptive vaginal ring: a global step forward. Lancet Global health. 2019;7(8):e986–7. 31231064 10.1016/S2214-109X(19)30289-X

[21] Division of AIDS (DAIDS) . Table for grading the severity of adult and pediatric adverse events version 2.1. March 2017 [cited 2020 Aug 10]. https://rsc.niaid.nih.gov/sites/default/files/daidsgradingcorrectedv21.pdf

[22] Division of AIDS (DAIDS) . Table for grading the severity of adult and pediatric adverse events addendum 1 female genital grading table for use in microbicide studies [cited 2020 Aug 10]. https://rsc.niaid.nih.gov/sites/default/files/addendum‐1‐female‐genital‐grading‐table‐v1‐nov‐2007.pdf

[23] Nel A , Bekker LG , Bukusi E , Hellstrӧm E , Kotze P , Louw C , et al. Safety, acceptability and adherence of dapivirine vaginal ring in a microbicide clinical trial conducted in multiple countries in Sub‐Saharan Africa. PLoS One. 2016;11:e0147743. 26963505 10.1371/journal.pone.0147743 PMC4786336

[24] Seserko LA , Emory JF , Hendrix CW , Marzinke MA . The development and validation of an UHPLC‐MS/MS method for the rapid quantification of the antiretroviral agent dapivirine in human plasma. Bioanalysis. 2013;5(22):2771–83. 24256358 10.4155/bio.13.256 PMC4104354

[25] Chen BA , Zhang J , Gundacker HM , Hendrix CW , Hoesley CJ , Salata RA , et al. Phase 2a Safety, pharmacokinetics, and acceptability of dapivirine vaginal rings in US postmenopausal women. Clin Infect Dis. 2019;68(7):1144–51. 30289485 10.1093/cid/ciy654 PMC6424088

[26] Parsons TL , Emory JF , Seserko LA , Aung WS , Marzinke MA . Dual quantification of dapivirine and maraviroc in cervicovaginal secretions from ophthalmic tear strips and polyester‐based swabs via liquid chromatographic‐tandem mass spectrometric (LC‐MS/MS) analysis. J Pharm Biomed Anal. 2014;98:407–16. 25005891 10.1016/j.jpba.2014.06.018 PMC4143664

[27] Lyndgaard L , Spangberg R , Gilmour C , Lyndgaard C . van den bBerg F. A process analytical approach for quality control of dapivirine in HIV preventive vaginal rings by Raman spectroscopy. J Raman Spectrosc. 2014;45(2):149–56.

[28] Baeten JM , Brown ER , Hillier SL . Dapivirine vaginal ring for HIV‐1 prevention. N Engl J Med. 2017;376(10):995–6. 10.1056/NEJMc1616546 28273023

[29] Nel A , van Niekerk N , Kapiga S , Bekker L‐G , Gama C , Gill K , et al. Safety and efficacy of a dapivirine vaginal ring for hiv prevention in women. N Engl J Med. 2016;375(22):2133–43. 27959766 10.1056/NEJMoa1602046

[30] Nel A , Haazen W , Nuttall J , Romano J , Rosenberg Z , van Niekerk N . A safety and pharmacokinetic trial assessing delivery of dapivirine from a vaginal ring in healthy women. AIDS. 2014;28(10):1479–87. 24901365 10.1097/QAD.0000000000000280

[31] Bunge KE , Levy L , Szydlo DW , Zhang J , Gaur AH , Reirden D , et al. Brief report: Phase IIa safety study of a vaginal ring containing dapivirine in adolescent young women. J Acquir Immune Defic Syndr. 2020;83(2):135–9. 31929401 10.1097/QAI.0000000000002244 PMC8577288

[32] Roberts ST , Nair G , Baeten JM , et al. Impact of male partner involvement on women's adherence to the dapivirine vaginal ring during a phase III HIV prevention trial. AIDS Behav. 2020;24(5):1432–42. 31667678 10.1007/s10461-019-02707-1 PMC7162727

[33] Montgomery ET , van der Straten A , Chitukuta M , Reddy K , Woeber K , Atujuna M , et al. Acceptability and use of a dapivirine vaginal ring in a phase III trial. AIDS. 2017;31(8):1159–67. 28441175 10.1097/QAD.0000000000001452 PMC5557083

[34] Laborde ND , Pleasants E , Reddy K , Atujuna M , Nakyanzi T , Chitukuta M , et al. Impact of the dapivirine vaginal ring on sexual experiences and intimate partnerships of women in an HIV prevention clinical trial: managing ring detection and hot sex. AIDS Behav. 2018;22(2):437–46. 29151197 10.1007/s10461-017-1977-1 PMC5866044

[35] Montgomery ET , Stadler J , Naidoo S , Katz AWK , Laborde N , Garcia M , et al. Reasons for nonadherence to the dapivirine vaginal ring: narrative explanations of objective drug‐level results. AIDS. 2018;32(11):1517–25. 29957723 10.1097/QAD.0000000000001868 PMC6230508

[36] Duby Z , Katz AWK , Browne EN , Mutero P , Etima J , Zimba CC , et al. Hygiene, blood flow, and vaginal overload: why women removed an HIV prevention vaginal ring during menstruation in Malawi, South Africa, Uganda and Zimbabwe. AIDS Behav. 2020;24(2):617–28. 31030301 10.1007/s10461-019-02514-8 PMC6815681

[37] Watnick D , Keller MJ , Stein K , Bauman LJ . Acceptability of a tenofovir disoproxil fumarate vaginal ring for hiv prevention among women in New York City. AIDS Behav. 2018;22(2):421–36. 29147810 10.1007/s10461-017-1962-8

[38] Roberts S , Hawley I , Luecke E , et al. Acceptability and preference for 3‐month versus 1‐month vaginal rings for HIV‐1 risk reduction among participants in a phase 1 trial: a mixed methods analysis. Paper presented at: 23rd International AIDS Conference Virtual; 2020 Jul 6‐10; San Francisco and Oakland, California. Abstract PEC0688. 10.1089/jwh.2021.0121 PMC9299526 34665672

[39] Montgomery ET , Beksinska M , Mgodi N , Schwartz J , Weinrib R , Browne EN , et al. End‐user preference for and choice of four vaginally delivered HIV prevention methods among young women in South Africa and Zimbabwe: the Quatro Clinical Crossover Study. J Int AIDS Soc. 2019;22:e25283. 31069957 10.1002/jia2.25283 PMC6506690

[40] van der Straten A , Browne EN , Shapley‐Quinn MK , et al. First impressions matter: how initial worries influence adherence to the dapivirine vaginal ring. J Acquir Immune Defic Syndr. 2019;81(3):304–10. 30844995 10.1097/QAI.0000000000002028 PMC6571014

